# A novel liver specific isoform of the rat LAR transcript is expressed as a truncated isoform encoded from a 5'UTR located within intron 11

**DOI:** 10.1186/1471-2199-10-30

**Published:** 2009-04-08

**Authors:** Simon JM Welham, Adrian JL Clark, Andrew M Salter

**Affiliations:** 1 University of Nottingham, Division of Nutritional Sciences, Sutton Bonington Campus, Loughborough, Leicestershire, LE12 5RD, UK; 2 Centre for Endocrinology, Barts & the London, Queen Mary University of London, UK

## Abstract

**Background:**

The leukocyte common antigen related receptor (LAR) protein has been shown to modulate the signal transduction of a number of different growth factors, including insulin and insulin-like growth factor 1. Splice variants exhibit differing roles and are expressed according to tissue type and developmental stage.

**Results:**

Using 5'RACE, we identified a 5'UTR within intron 11 of the rat LAR gene. We demonstrated that this gives rise to a novel isoform of the LAR transcript encoded from the identified region within intron 11. By priming across the site from exon 11 to exon 15 we show that the novel 5'UTR is not represented in the full-length transcript and thus, it produces a truncated form of the LAR mRNA. We examined the tissue distribution of this novel isoform and found it to be exclusively expressed in liver. We additionally identified a liver specific 150 kDa band with western blotting which we propose may represent the protein product of the novel transcript. Luciferase assays showed the region immediately upstream of the 5'UTR to possesses considerable promoter activity and that this may be conferred by the presence of a number of putative binding sites for liver enriched transcription factors.

**Conclusion:**

In summary, we describe a novel, liver specific, truncated isoform of the LAR transcript transcribed under the control of an intronic promoter, potentially representing a previously unidentified modulator of hepatic insulin signalling.

## Background

The leukocyte common antigen related receptor (LAR) is a receptor protein tyrosine phosphatase which has been identified as a modulator of insulin and insulin-like growth factor 1 (IGF1) signal transduction. In addition, it has been widely studied as a key component of neural development. The LAR protein contains 3 immunoglobulin-like (Ig) and 8 fibronectin type III-like (FN) domains extracellularly, a transmembrane domain (TM) and 2 intracellular phosphatase domains (PTP-1 and PTP-2). The extracellular domains mediate cell-cell and cell-extracellular matrix (ECM) interactions. Modulation of signal transduction pathways occurs via its phosphatase activity which is conferred by the first phosphatase domain [1,2]. The second is important for substrate recognition.

The expression of up to 32 different isoforms of the LAR transcript have been postulated to result from 5 separate splicing variations [3] within the fibronectin-like domains, proximal to the transmembrane domain and at the 3' end of the transcript. Different isoforms of the LAR transcript have been shown to be differentially expressed across a wide range of tissues [3], during different stages of neural development [4] and a specific isoform containing a distinct ectodomain in the fifth FNIII domain has been found to be important in the promotion of neurite outgrowth [5].

The LAR induced modulation of neural growth appears to be mediated by its interaction with the nerve growth factor (NGF) [6] and brain derived neurotrophic factor (BDNF) [7] signalling pathways, whilst its influence on the insulin signalling pathway may play a significant role in glucose homeostasis. This variety of functions across different tissue types suggests either a commonality of structural interaction, or a tissue specific variation in isoform expression.

Through modulation of the insulin signalling pathway the LAR protein has been shown to promote an insulin resistant phenotype. The LAR protein physically interacts with the insulin receptor (IR) [8] and promotes its de-phosphorylation [9,10] via its cytoplasmic domains [11]. Direct interaction between the LAR protein and IR was seen to be increased with insulin treatment [8]. Antisense knockdown of LAR transcript *in vitro *leads to a marked elevation of IR autophosphorylation and insulin dependent phosphatidylinositol 3-kinase (PI3K) activity [12-14], whilst Chinese hamster ovary (CHO) cells overexpressing LAR protein show a reduction of insulin receptor (IR) and insulin receptor 1 (IRS1) phosphorylation [10].

Here we identify a novel, liver specific isoform of the rat LAR transcript encoded from a 5'UTR located within intron 11 of the full-length gene and examine the expression of this isoform in a rat model of insulin resistance induced by fetal exposure to a maternal dietary protein restriction [15].

## Results

### 5'RACE identified a novel 5'UTR within the liver expressed rat transcript

**Figure 1:**
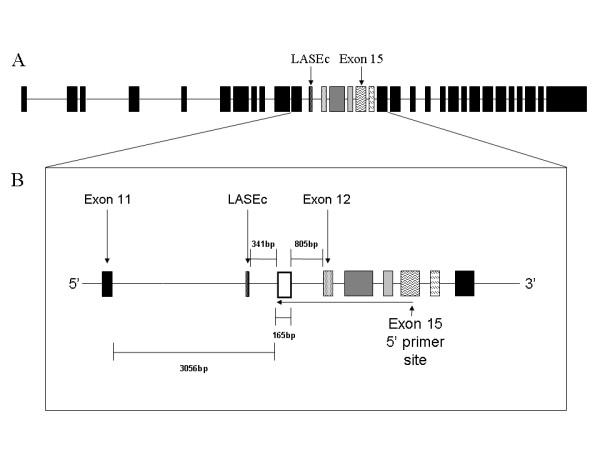
**5'RACE identification of a novel 5'UTR in intron 11 of the LAR gene**. (A) Structure of the rat LAR gene. (B) Magnification of the region of the novel 5'UTR. Open box – 5'UTR, LASEc – LAR alternatively spliced element c. Exons and alternatively spliced elements are represented as boxes in all figures, labelled as follows: White dots on black background – LASEc, black dots on white background – exon 12, dark grey – exon 13, light grey – exon 14, wavy lines – exon 15 & diagonal dashing – exon 16.

We carried out a 5'RACE analysis of the LAR transcript, priming from within exon 15. This identified a unique transcript including 165 bp of intron 11 located 805 bp upstream of exon 12 and 3056 bp downstream of exon 11, in addition to exons 12–15 (Figure 1). The identified region was also located 341 bp downstream of a previously described LAR alternatively spliced element (LASEc; [3]). BLAST analysis of this region against mouse and human sequences failed to show the presence of any un-annotated exons, demonstrating that we had identified either, a novel 5'UTR for a truncated transcript coding from this region or an unidentified exon.

### A shortened transcript is encoded from the new 5'UTR

**Figure 2:**
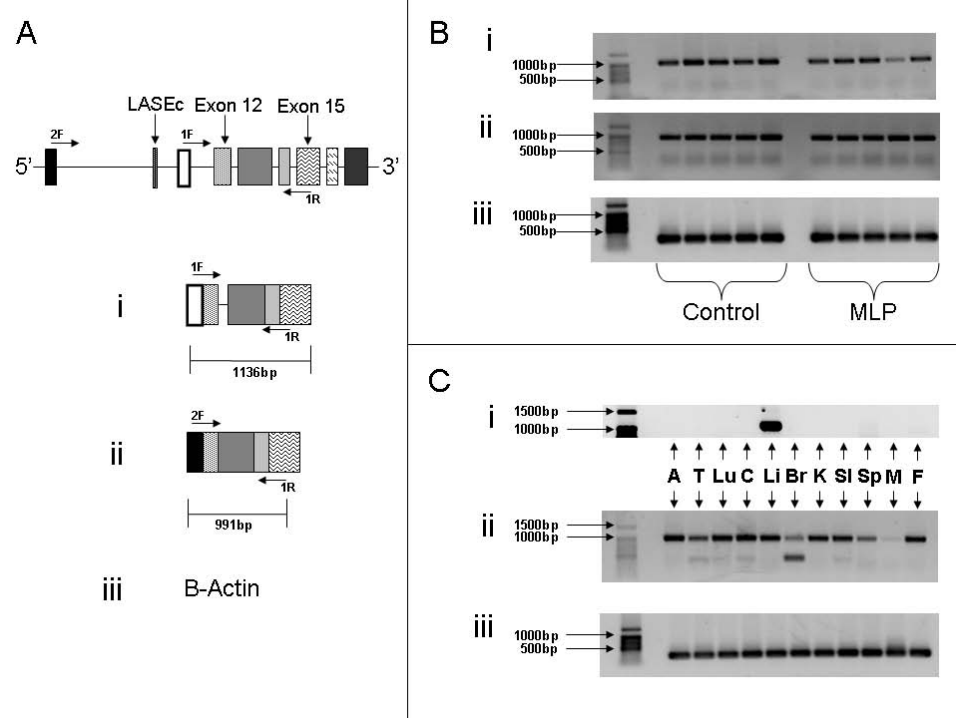
**PCR analysis of the novel transcript from a variety of rat tissues**. (A) Primer locations to produce either (i) the novel transcript, (ii) the full-length transcript spanning the region of the 5'UTR, or (iii) beta-actin. These primers were used to examine the presence of the novel transcript in (B) liver from male offspring of rats supplied either a control (Control) or low protein diet (maternal low protein-MLP) during gestation and lactation and (C) a variety of normal rat tissues. A-Adrenal, T-Testis, Lu-Lung, C-Colon, Li-Liver, Br-Brain, K-kidney, SI-Small Intestine, Sp-Spleen, M-Muscle, F-Fat.

In order to confirm the presence of this putative 5'UTR within the native mRNA, we carried out PCR analysis of cDNA derived from rat liver. Primers were designed to recognise a region within the newly identified 5'UTR and a region of exon 15 (Figure 2A). PCR products of 1061 bp were expected, and bands of over 1 kb were observed (Figure 2B). Sequencing confirmed the presence of the 5'UTR and exons 12–15 within the novel transcript, however, we also noted the exclusive retention of intron 12 (75 bp), thus producing a band of 1136 bp (Figure 2B). The observation of a retained intron within the PCR product which had not been seen in the 5'RACE suggests that different forms of this novel transcript may exist.

We chose to examine the expression of the newly identified isoform in liver tissue from animals exposed to a maternal dietary protein restriction during gestation (Figure 2Bi) in order to determine the contribution, if any, of the LAR gene product in the development of insulin resistance in this model of metabolic programming. Beta-actin was used as a loading control (Figure 2Biii). From gross analysis of the PCR products, we did not see any overt differences in expression levels of any of the products described above, between animals exposed to maternal undernutrition during gestation when compared with controls. We therefore chose not to examine the expression levels in any more detail in these animals.

To demonstrate that the novel 5'UTR was exclusively part of a transcript encoded from this point downstream, rather than merely an additional exon of the full-length transcript, we carried out PCR analysis of rat liver cDNA using primers designed against exons 11 and 15 (Figure 2Bii). The expected band of 991 bp was observed with no other bands visible, supporting the hypothesis that the newly identified internal 5'UTR was indeed exclusively part of a transcript encoded from this point.

### The newly identified transcript is expressed exclusively in liver tissue

We examined expression of the LAR transcript and the newly identified isoform in a number of rat tissues in order to determine its distribution. cDNA was synthesised from RNA extracted from adrenal, testis, lung, colon, liver, brain, kidney, small intestine, spleen, skeletal muscle and fat, from an adult male rat supplied standard laboratory chow and tested for the presence of the newly identified transcript. We found expression of the new isoform was exclusively confined to liver (Figure 2Ci) whilst the transcript identified using primers against exons 11 and 15 was observed in all tissues tested (Figure 2Cii). This further confirmed that the newly identified 5'UTR represents the first exon of a truncated transcript encoded from this point as it did not appear in native transcripts which include the upstream exon 11.

We observed strong expression of the "full-length" transcript (i.e. those including exon 11) in all tissues except muscle and brain, which showed lower levels of this isoform. Instead, at least in brain, LAR mRNA was significantly represented by a shortened isoform of the "full-length" transcript. This shortened isoform was also observed, albeit to a much lesser extent, in testis, colon and small intestine (Figure 2Cii). Beta-actin was used to demonstrate equivalent loading (Figure 2Ciii).

Using real-time PCR we found that the full transcript was expressed at a level approximately 2 to 3 fold higher than that of the truncated hepatic isoform.

**Figure 3:**
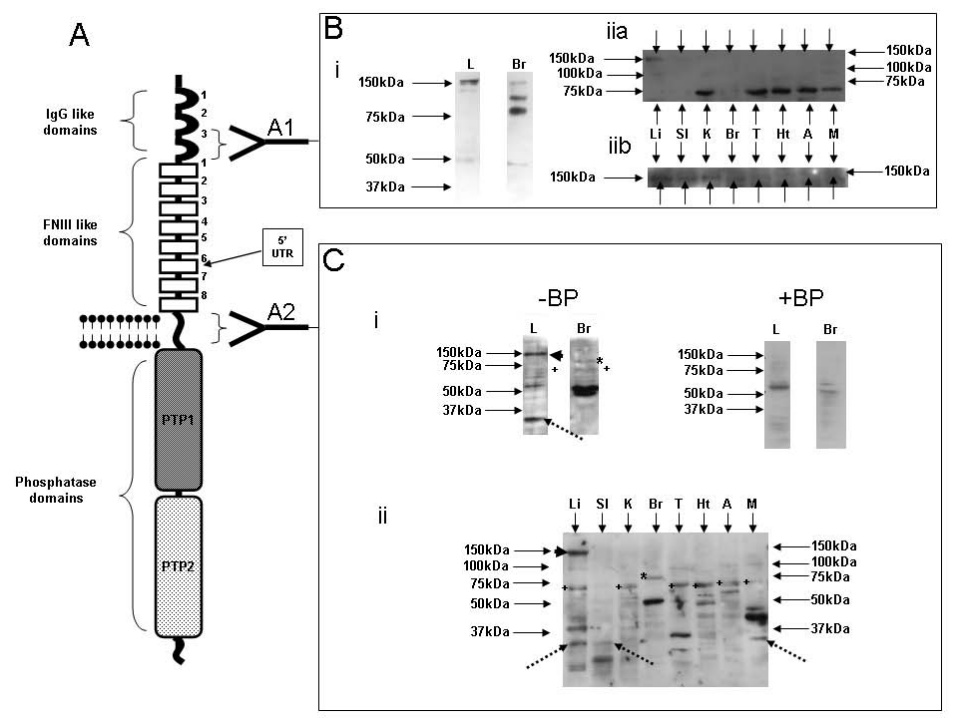
**LAR protein expression in liver and brain**. (A) Full-length LAR protein structure showing the location of the IgG-like, FNIII-like, transmembrane and phosphatase domains. The target regions of antibodies A1 and A2 are shown. The location of the putative translation start site of the novel isoform encoded by the 5'UTR is also shown. (Bi) Western blot of liver (L) and brain (Br) tissue probed using antibody A1. (Biia) Western blot of liver (Li), small intestine (SI), kidney (K), brain (Br), testicle (T), heart (Ht), adrenal (A) and muscle (M) probed using antibody A1. Arrows identify the E-subunit. (Biib) A lengthened exposure of the western blot shown in Biia identifying the 150 kDa E-subunit in all tissues (arrows). (Ci) Western blot of liver and brain probed with antibody A2 either without (-BP) or with (+BP) blocking peptide. (Cii) Western blot of liver, small intestine, kidney, brain, testicle, heart, adrenal and muscle using antibody A2. Arrowhead identifies a liver specific band at 150 kDa. Dotted arrow identifies a band at 30 kDa. * identifies the P-subunit, + identifies the LAR-CTF.

In order to determine whether a protein product of the truncated isoform might be produced, we carried out Western blotting using two different antibodies, one directed upstream of the region encoded by the transcript subsequent of the 5'UTR (amino acids 24–196 of human LAR; antibody A1; Figure 3A) and another targeted downstream of this site (amino acids 1250–1350 of human LAR; antibody A2) in a panel of tissues. Probing with antibody A1 initially in liver and brain identified a single band of approximately 150 kDa in both tissues, representing the processed extracellular region of the full-length protein described previously as the E-subunit (Figure 3Bi; [16]), in addition to two shorter bands of approximately 100 kDa and 80 kDa in brain. Probing liver and brain using antibody A2 revealed a different pattern of bands (Figure 3Ci -BP). In liver, we detected bands at approximately 150 kDa, 72 kDa, 70 kDa, 60 kDa and 30 kDa, whilst in brain, we observed bands at approximately 85 kDa, 72 kDa, 70 kDa, 60 kDa and 50 kDa. The bands seen at 85 kDa, 72 kDa and 70 kDa most likely represent the internal processed P-subunit, the LAR c-terminal fragment LAR-CTF and the LAR intracellular domain LICD respectively [16,17], demonstrating the ability of this antibody to specifically identify the LAR protein. With the exception of the bands at ~60 kDa, all of the species described were considerably diminished in intensity or disappeared altogether when blocking peptide was incubated with the primary antibody (Figure 3Ci +BP). This additionally suggests that these bands may well represent specific LAR protein targets of the antibody whilst the ~60 kDa bands may well be non-specific targets of antibody A2. It was interesting to note two additional bands in brain tissue at approximately 50 kDa with antibody A2. Non-specific proteolysis is most likely to occur in brain tissue due to the rapid rate of its degradation post-mortem, however, the lack of further, shorter bands in brain tissue, in addition to the lack of any short bands using antibody A1, might suggest that these two are representative of either different brain specific isoforms, or novel processed forms of the full-length protein. It should be noted, that these bands also diminished significantly after incubation with blocking peptide (Figure 3Ci +BP).

We therefore identified two apparently liver specific bands of 150 kDa and 30 kDa using an antibody directed downstream of the novel transcription start site. Antibody A2, as previously mentioned, was raised against a peptide corresponding to amino acids 1250–1350 of the human full-length LAR protein (aa1241-1341 in the rat protein). The LAR protein is processed in vivo via several cleavage events. The 150 kD E-subunit is produced by processing at a furin cleavage site located immediately below amino acid 1164 (1173 in the human) with the 85 kD P-subunit comprising the region downstream of this site. The 150 kDa band identified in liver by antibody A2 therefore cannot be the E-subunit as the furin cleavage site is upstream of the region against which the antibody was raised and thus may represent a unique isoform of the LAR protein.

In order to determine whether the 150 kDa and 30 kDa isoforms were truly liver specific, we probed a wider panel of tissues using antibody A2 (Figure 3Cii). Most tissues examined demonstrated expression of the 72 kDa LAR-CTF (Figure 3Cii – +) and brain tissue was again shown to express the 85 kDa P-subunit (Figure 3Cii – *). This antibody identified numerous bands in different tissues, some of which may well be a consequence of non-specific binding. However, we found that the 150 kD band (arrowhead) is indeed liver specific, whilst the 30 kD protein (dotted arrows) is present in liver, muscle and, at very low levels, small intestine. We therefore suggest, that the 150 kD band identified by antibody A2 represents the likely protein product of the liver specific transcript which we have identified within this article. It should be noted that the E-subunit was demonstrated to be present in all tissues examined (Figure 3Biia and 3Biib – arrows) using antibody A1, confirming that the 150 kDa band identified with antibody A2 was not simply a consequence of a cross reaction with the E-subunit.

We have hypothesised that the methionine at position 817 of the LAR protein may represent the start of translation of the novel isoform. A protein including all downstream amino acids is estimated (using the BioEdit program – ) to be approximately 123 kD in size. It should be noted that BioEdit predicts the sequence encoding the E-subunit of the full-length LAR to be approximately 129 kD in size. This would support the contention that the 150 kDa band, does indeed represent the protein product of the novel liver specific transcript.

### The 5'UTR and the upstream region show species conservation

**Figure 4:**
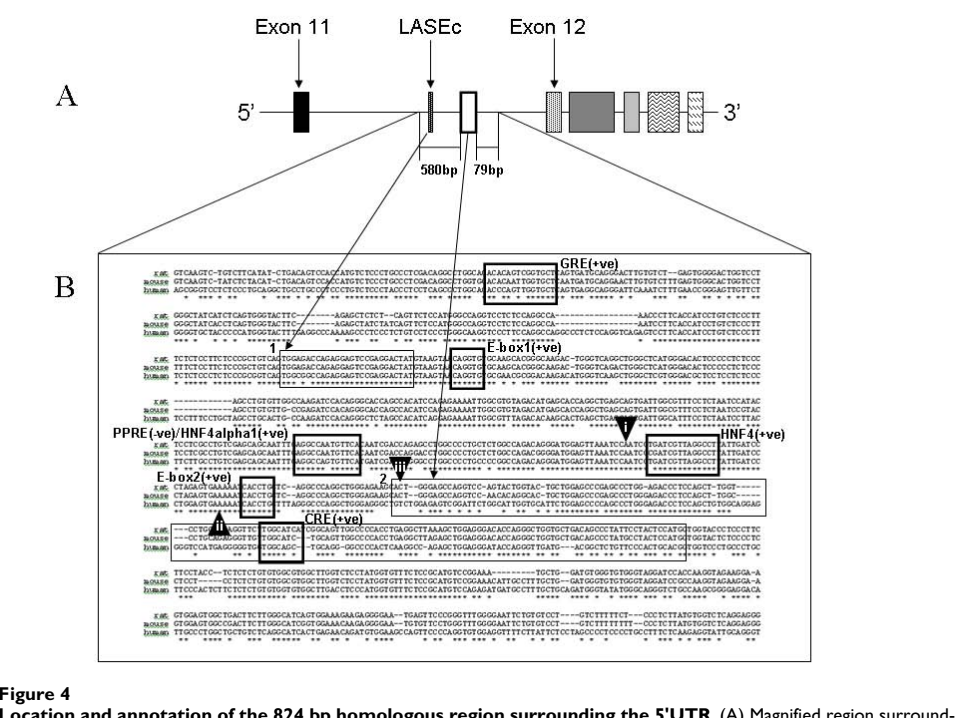
**Location and annotation of the 824 bp homologous region surrounding the 5'UTR**. (A) Magnified region surrounding the 5'UTR. (B) Rat, mouse and human sequences corresponding to this region. Thin boxes correspond to the (1) LASEc site and the (2) novel 5'UTR. Thick boxes correspond to putative transcription factor binding sites-GRE-Glucocorticoid response element, PPRE-peroxisome proliferator activated receptor response element, HNF4α-Hepatocyte nuclear factor 4 alpha, CRE-cAMP response element. +ve = located on the sense strand; -ve = located on the antisense strand. Arrowheads correspond to (i) CAAT box, (ii) putative TATA box and (iii) transcription start site.

We proposed that, if this region represents a conserved transcriptional initiation site for a shortened transcript, then it should share sequence homology with other species. The intronic region containing the 5'UTR was subject to BLAST analysis against mouse and human sequences. The regions identified (Figure 4A) were then examined using the ClustalW sequence alignment tool and those regions of closest homology determined.

We found an 824 bp conserved region of intronic DNA which contained the 5'UTR and extended 582 bp upstream encompassing the previously described LASEc, plus an additional 212 bp 5' of LASEc in addition to 79 bp downstream of the 5'UTR (Figure 4A&4B). This region showed sequence homology of 95% with mouse and 82% with human. Comparison of the sequence between LASEc and the start of the 5'UTR showed sequence homology of 97% between rat (341 bp) and mouse (340 bp) and 88% between rat and human (355 bp).

Comparison of the remaining portion of the upstream intron demonstrated sequence homology of 85% between rat and mouse and 53% between rat and human. Downstream of the consensus region, the intronic sequence homology between rat and mouse was 70% whilst that between rat and human was only 56%.

For the sake of completeness, we also examined the sequence homology between species of the upstream exon (97% between rat and mouse, 88% between rat and human) and the downstream exon (94% between rat and mouse, 88% between rat and human).

### The region upstream of the 5'UTR demonstrates promoter activity

The considerable degree of homology across species supported the hypothesis that this region represents a conserved promoter. Furthermore, closer examination of the promoter region revealed the presence of putative transcription factor consensus binding sites which may be important in determining its liver specific expression (Figure 4B). We identified two E-boxes, two hepatocyte nuclear factor 4 alpha (HNF4α) binding sites, a peroxisome proliferator-activated receptor response element (PPRE) and a cAMP response element (CRE). The PPRE and the first HNF4α consensus sites overly each other, with the PPRE on the "antisense" (-ve) strand and the HNFα site on the "sense" (+ve). We also observed a putative glucocorticoid response element upstream of the LASEc containing region.

We further identified a putative CAAT box 72 bp upstream of the transcription start site (TSS) in addition to a potential TATA binding protein (TBP) binding site located 37 bp upstream of the TSS (Figure 4B).

**Figure 5:**
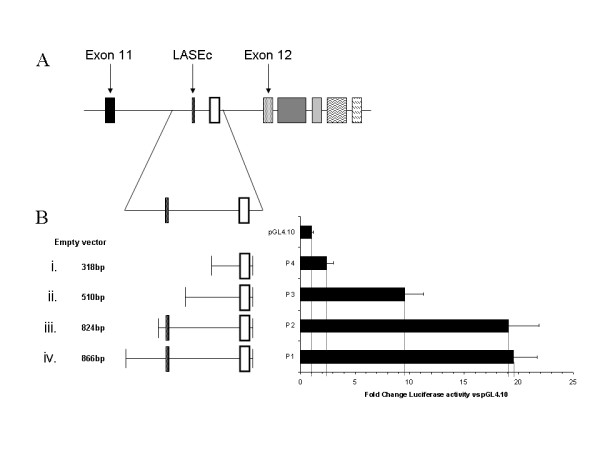
**Promoter activity of the region surrounding the 5'UTR**. (A) Region around the 5'UTR used for the creation of promoter constructs. (B) Promoter constructs with corresponding luciferase activity.

We examined the activity of this putative promoter region using a luciferase assay. Regions around the 5'UTR (Figure 5) were amplified, cloned into the pGL4.10 luciferase expression vector and transfected into McArdle RH7777 cells.

Promoter constructs containing the complete region of homology in addition to 42 bp upstream (Figure 5Biv) demonstrated clear promoter activity in McArdle RH7777 cells. Loss of the 42 bp had no effect on promoter activity as values from the full-length construct were identical to those without the additional bases (P = 0.851; Figure 5Biii). The full-length construct showed an approximately 20 fold increase in promoter activity over baseline values from pGL4.10 [luc] alone (P < 0.01). Removal of the region containing LASEc and 75 bp downstream which includes the first putative E-box (Figure 5Bii), halved the activity of the promoter region (P < 0.01), but still retained considerable activity relative to the empty vector (approximately 10 fold higher; P < 0.01). The loss of an additional 192 bp (inclusive of the first HNF4α/PPRE site) almost completely abolished promoter activity (Figure 5Bi).

These data demonstrate that the 824 bp homologous region possesses considerable promoter activity conferred by two distinct parts. First, the region immediately upstream of the 5'UTR and secondly, by the region containing LASEc.

## Discussion

In this study we have demonstrated the existence of a novel, liver-specific transcript of the rat LAR gene. We identified a shortened isoform coding from an intronic region upstream of exon 12 of the LAR gene. This is produced under the control of an internal promoter region located within intron 11 and which includes a previously identified LAR alternatively spliced element (LASEc; [3]). We have shown that the region identified as a putative internal 5'UTR is not present within PCR products produced by priming between exons 11 and 15, indicating that this region is not contained within the full-length transcript and therefore, that it represents the first exon of a truncated form of the gene. Furthermore, expression of the newly defined transcript is exclusively confined to the liver.

A role for the LAR protein has been implicated in numerous biological processes ranging from neural development to modulation of insulin signalling [18]. Many isoforms have been identified which are expressed specifically according to both tissue type and developmental stage. The LAR protein has been implicated in the modulation of the signalling pathway of a number of growth factors, including nerve growth factor (NGF) [6], brain derived neurotrophic factor (BDNF) [7], insulin [8,10,12-14] and IGF1 [19].

These studies suggest a variety of roles for the LAR protein in the developing and postnatal individual which may be modulated by isoform, tissue type and pathological phenotype. We have demonstrated that expression of the shortened transcript is restricted to liver, with no evidence of expression in any other adult tissue despite the apparent widespread distribution of the full-length transcript. This would suggest that the control of expression of the newly identified isoform is under the control of liver enriched transcription factors. In addition, the liver restricted expression suggests a liver specific function for this isoform. With regard to postnatal liver function, of most relevance would be the insulin and IGF1 pathways, we therefore hypothesise that one or both of these signalling pathways may be influenced by the newly identified isoform.

Overexpression of LAR protein has been shown to depress IR and IRS1 phosphorylation in CHO cells [10] whilst skeletal muscle specific overexpression in the mouse, decreases glucose disposal and results in insulin resistance [20] apparently mediated by reduced IRS2 phosphorylation and IRS induced PI3K activity. In humans, obesity was shown to result in a significant elevation of LAR expression in skeletal muscle [21] in association with considerably impaired insulin induced glucose disposal and enhanced phosphatase activity. Phosphatase activity was reduced to levels seen in lean individuals only by immunodepletion of LAR protein.

LAR transcript knockdown in McArdle RH7777 rat hepatoma cells [12-14] leads to a marked elevation in IR autophosphorylation, whilst a similar loss in HEK293 cells appears to have no effect on IR or IRS1 phosphorylation but instead, results in post-receptor insulin resistance as evidenced by impaired Akt and MAPK phosphorylation [22]. Loss of LAR gene product in a mouse knockout model results in reduced fasting levels of insulin, glucose [23] and IGF-1 [19] alongside elevation of IGF-1 stimulated phosphorylation of IGF-1R, IRS1, IRS1 associated PI3K and extracellular signal related kinase (ERK1/2) activity in vascular smooth muscle cells. In these same animals, absence of LAR gene product is associated with a greatly enhanced proliferation of the neointima in response to arterial injury. The functional role of the LAR protein appears, therefore, highly tissue specific.

The transcription start site (TSS; Figure 4B arrowhead iii) is preceded by two structures indicative of a classical promoter, namely a CAAT box (arrowhead i) and a putative TATA box (arrowhead ii). The CAAT box is located 73 bp upstream of the TSS whilst the sequence TGAAAA which may bind the TATA box binding protein (TBP) is located in an appropriate position 37 bp upstream of the TSS. The promoter region appears to contain consensus binding sites for a number of transcription factors relevant to the isoform's liver specific expression profile. Of note are two HNF4α binding sites, a PPRE and two E-boxes. All of the putative binding sites share good homology (100% in the case of the E-boxes) with mouse and human (Figure 4). The activity of the promoter was shown to be significantly reduced with the loss of the sequence containing the first E-box (Figure 5Bii) and was further diminished with the removal of the first HNF4α/PPRE site (Figure 5Bi). The low level of activity of the shortest promoter construct suggests that either the response elements identified within this region (the second HNF4α, second E-box and CRE) are inactive, or that their activity is driven by specific cellular responses.

The presence of HNF4α binding sites may imply constitutive expression of the shortened isoform in liver, however, the additional E-boxes suggests the possibility of nutritional control of expression, particularly if the steroid response element binding protein 1c (SREBP1c) is found to transactivate the promoter at either or both of these sites. SREBP1c activity is elevated in response to glucose and insulin signalling [24] and thus, expression control exerted by this protein might underlie a novel feedback loop with the insulin signalling pathway.

We examined the expression of the novel isoform of the LAR transcript in an established model of developmentally programmed adult insulin resistance [15] in order to identify whether this specific transcript may play a role in the development of the insulin resistant phenotype. However, we failed to observe any overt differences in expression of the newly identified shortened isoform between males from either dietary group and thus chose not to pursue this further.

In the rat, the LAR gene is encoded by 31 exons to produce a protein possessing 3 IgG like domains, 8 FNIII-type domains, a transmembrane domain and two intracellular phosphatase domains (Figure 3A) [1]. The transcription start site of the shortened isoforms described here is located towards the end of the 5^th ^FNIII type domain and therefore it is predicted that the protein product of this isoform would only possess a limited extracellular domain. The first ATG encountered when examining the truncated transcript actually exists at the end of the novel 5'UTR and would be in frame with the remainder of the transcript. However, closer inspection of the homologous region in the human sequence shows this site to be represented as ACG (Figure 4B) and therefore the rat ATG is likely to be a consequence of a de-amination event [25,26] in early rodent evolution (as the ATG is present in the mouse sequence also). It is, therefore, likely that the initiator methionine is encoded by the first of two adjacent ATG codons located within exon 13. This methionine is close to the start of the FNIII-6 domain (amino acid 817 of the full-length LAR protein) as predicted by Pfam analysis of the protein sequence and thus, it is anticipated that the protein product of this truncated isoform should possess the majority of the FNIII-6 domain in addition to an unknown portion or portions of the protein downstream of this site. Examination of the region around the predicted initiator methionine (CCACC**ATG**A) shows tight homology with the classical Kozak sequence (CCA/GCC**ATG**G/A; [27,28]). The +4 position relative to the start of the ATG is most commonly represented by a G, however the next most frequent occurrence is an A residue. This, alongside the presence of an A in the -3 position, supports the assertion that the transcript encoded downstream of the novel 5'UTR is translated.

Importantly, we identified a liver specific band with Western blotting of approximately 150 kDa which we propose may represent the translated product of this truncated transcript. If, as we have suggested, translation begins with the methionine at position 817, then this would result in the production of a protein which included part of the FNIII 6 domain in addition to domains FNIII 7 & 8, as well as the transmembrane domain and both phosphatase domains. Thus, such a protein should retain the capacity to de-phosphorylate target proteins. Examination of the region immediately downstream of this methionine for the presence of a signal peptide using the SignalP 3.0 server [29,30] failed to identify a signal sequence, possibly suggesting that the protein may not be secreted and retained within the cytoplasm, but this does not preclude its capacity to be trafficked to the plasma membrane. The truncation of the extracellular domain may influence the control of activity of this novel isoform as homophilic binding to extracellular regions (FNIII 3) with an ectodomain isoform or a peptide mimetic of this isoform directly influences LAR phosphatase activity during neural development [5,31]. The modification of the extracellular domain of this isoform may therefore influence LAR protein activity in a different manner to that of the full-length protein.

Interestingly, the 5^th ^FNIII domain appears to be important in the mediation of LAR protein function. A short ectodomain isoform of the LAR protein containing a novel shortened amino terminus in addition to the FNIII-5 domain (LARFN5C) was shown to act as a direct ligand of the LAR protein and enable neurite outgrowth [31] as was a peptide mimetic complementary to this region [5]. It has been suggested that the activity of receptor protein tyrosine phosphatases (rPTP) is regulated by homophilic interactions [32], with monomeric rPTPs being constitutively active and dimerization leading to inactivation. Thus, it was proposed that the activity of the LAR protein induced by binding of the LARFN5C isoform or mimetic peptide, might be mediated by preventing inactivation through homophilic binding. It is noteworthy that LAR protein activity induced by ECM interactions, in particular with the laminin-nidogen complex, appears similarly to be mediated via the FNIII-5 domain [33]. It remains to be seen what the nature of the complete transcript and its protein product is, in addition to the protein function and whether this is exerted intra- or extracellularly.

In summary, we have identified a novel isoform of the LAR transcript which is encoded from a region within intron 11 and is exclusively expressed in liver. This isoform is under the control of a promoter located upstream of the newly identified 5'UTR. Future work will aim to understand the functional significance of hepatic expression of this new truncated isoform, in particular in relation to insulin signal transduction, in addition to the control of its expression.

## Conclusion

The LAR gene product is expressed in a number of isoforms and this is dependent upon tissue type. It would appear that the functional role of the LAR protein may be principally determined by the isoform expressed and in particular, by the structure of the region around the FNIII domains 4–7. Thus it may be suggested that tissue specific function is conferred by the nature of the extracellular region of the LAR protein. We have identified a novel truncated isoform of the LAR transcript which we have further demonstrated to be liver specific and which is expressed under the control of a promoter region possessing consensus sites for a number of liver enriched transcription factors. We propose that this represents a novel mechanism for the determination of liver specific LAR protein function which, by virtue of the restriction of its expression, may prove to play a significant role in the modulation of the hepatocyte response to insulin signalling.

## Methods

### Animals

All animal work was carried out in accordance with the Scientific Procedures (Animals) Act 1986, UK. Female Wistar rats (Charles River, UK) were mated and supplied either a control diet containing 20% protein by weight (Control) or a low protein diet containing 8% protein by weight (MLP – Maternal Low Protein) throughout pregnancy and lactation. At birth, offspring were randomly culled to 8 per litter (4 males and 4 females) and then weaned onto identical diets at 3 weeks of age. At 4 weeks of age, animals were fasted for 12 hours and sacrificed using schedule 1 methods. Organs were removed and snap frozen in liquid nitrogen and subsequently stored at -80 C for later analysis. Up to 3 litters were used from each dietary group.

### RNA ligase-mediated rapid amplification of 5' cDNA ends

5'RACE was carried out using the Generacer Kit (Invitrogen, Paisley, UK). All procedures were carried out according to the manufacturer's instructions. 5'RACE nested PCR reactions were carried out using primer 5'1 targeted against exon 15 (GCAGTGCGGATGGACACCAGGTGCTGTA) for the first round and primer 5'2 located 41 bp upstream of primer 5'1 (GGTTCATCAGGACGAAGGAG), for the second round. PCR generated products were cloned using the TOPO TA cloning kit for sequencing (Invitrogen, Paisley, UK) and sequenced (Geneservice, Nottingham, UK).

5'RACE generated sequences were identified using the Basic Local Alignment and Search Tool provided by Ensembl . Sequences were searched against mouse and human genomes and regions found to be similar between the three species were compared using ClustalW version 1.83 .

### Promoter constructs

Regions surrounding the newly identified 5'UTR were amplified from template DNA produced by PCR using primers upstream (GCTGAGAGCAGGATGGGTAG) and downstream (CACTGATGCCCAAGAAGTCA) of the site of interest (1039 bp). Constructs were produced using primers designed to insert an XhoI consensus site 5' and an EcoRV site 3' of the DNA product for subsequent digestion. Constructs were produced in order to enable the determination of promoter activity of the entire homologous region upstream of the newly identified 5'UTR. Therefore a reverse primer, directed against the sequence 80 bp downstream of the new 5'UTR (ATATGGATATCTTTCCGGACATGCGGAGAAA), was used in PCR against four different forward primers. The forward primers used produced constructs of 318 bp (ATATTCTCGAGTCCAATCCTGATCGTTAGGC), 510 bp (ATATTCTCGAGGTGTTGGCCAAGATCCACAG), 824 bp (ATATTCTCGAGCTGACAGTCCACCATGTCTC) and 866 bp (ATATTCTCGAGAGTGTCTTCCCTCTGCCTGA) respectively. All constructs and pGL4.10 [luc2] expression vector were cut first with EcoRV (Promega, Southampton, UK) and second with XhoI (Promega, Southampton, UK) restriction endonucleases according to manufacturers guidelines. DNA constructs were then ligated into digested pGL4.10 [luc] using T4 DNA ligase (NEB, Hitchin, UK) according to manufacturers instructions. Ligated vectors were then cloned into JM109 competent *E. Coli *(Promega, Southampton, UK).

### Cell culture and luciferase assay

McArdle RH7777 rat hepatoma cells were grown in DMEM (Sigma, Poole, UK) supplemented with Penicillin/Streptomycin (Sigma, Poole, UK), 2 mM L-Glutamine (Sigma, Poole, UK) and 10% heat inactivated fetal bovine serum (Gibco). Cells were plated into 24-well tissue culture plates (Falcon) at a density of approximately 100,000/well. Promoter constructs were transfected into McArdle RH7777 cells using Fugene HD (Roche Diagnostics, West Sussex, UK) according to the manufacturers instructions. After 48 hrs, cells were lysed using luciferase substrate (Promega, Southampton, UK) and lysates were analysed for luciferase activity with a microplate luminometer (Turner Designs, California, USA). All assays were carried out in triplicate.

### PCR analysis of transcripts

PCR was carried out in order to verify the presence of the newly identified 5'UTR in native mRNA. Primer locations are annotated in Figure 2A. One forward primer directed against the 5'UTR (1F; TGGGAGCCAGGTCCAGTACT) was used with a reverse primer directed against exon 15 (1R; GGTTCATCAGGACGAAGGAG) to produce a product of 1137 bp. A forward primer directed against exon 11 (2F; ATTCGTGGCTACCAGGTCAC) was used against 1R to demonstrate that the new 5'UTR was not incorporated within the full-length transcript (991 bp). Primers directed against rat β-actin were used to show equal loading (251 bp; Forward – AGTACCCCATTGAACACGGC, Reverse – AATGCCAGTGGTACGACCAGA). The relative expression levels of the full-length transcript and novel truncated isoform were compared using real-time PCR. Primers were designed to detect the full transcript (138 bp; Forward – ATTCGTGGCTACCAGGTCAC, Reverse – AATGGAGTAGGTGGTCTCGG) and the novel isoform (135 bp; Forward – CACCTGAGGCTTAAAGCTGG, Reverse – CCCTTAGTGGTGTAGGCAGC). Real-time PCR was carried out in triplicate using Lightcycler 480 SYBR Green 1 Master (Roche Diagnostics, West Sussex, UK) and was run on a Lightcycler 480 (Roche Diagnostics, West Sussex, UK). All PCR products were sequenced to confirm their identity.

### Western blotting

Tissues were removed and immediately disrupted in 5 volumes of ice-cold homogenisation buffer (150 mM NaCl, 50 mM HEPES, 2.5 mM EDTA, 10% glycerol, 1% Triton, 1 mM Na_3_VO_4_, 10 mM NaF) containing a protease inhibitor cocktail (Roche Diagnostics, West Sussex, UK). Samples were homogenised on ice using a dounce homogeniser and subsequently passed 10 times through a 21 guage needle. Samples were spun at 13000 rpm at 4°C for 5 minutes and the supernatant removed. Protein concentration was determined using the Bio-Rad protein assay system (Bio-Rad, Hemel Hempstead, UK) according to manufacturer's instructions.

Approximately 50 μg protein was electrophoresed down a 10% polyacrylamide gel and transferred to nitrocellulose paper for probing with antibodies directed against different regions of the LAR protein (BD, Oxford, UK – antibody A1 and Abcam, Cambridge, UK – Antibody A2) Both primary antibodies were incubated at a concentration of 1:1000 in blocking solution (5% dried skimmed milk in tris-buffered saline with 1% Tween 20). Secondary antibodies conjugated to horseradish peroxidise (Sigma, Poole, UK; GE healthcare, Amersham, UK) were incubated at a concentration of 1:5000 in blocking solution. Bands were visualised using ECL Plus reagent (GE healthcare, Amersham, UK).

### Statistical analysis

Students T-test was used.

## Authors' contributions

SJMW participated in the study design, data interpretation, carried out all experimental work and wrote the manuscript. AMS participated in the study design, data interpretation and manuscript revision. AJLC participated in data interpretation and manuscript revision.
